# Digital templates and brain atlas dataset for the mouse lemur primate

**DOI:** 10.1016/j.dib.2018.10.067

**Published:** 2018-10-25

**Authors:** Nachiket A. Nadkarni, Salma Bougacha, Clément Garin, Marc Dhenain, Jean-Luc Picq

**Affiliations:** a Centre National de la Recherche Scientifique (CNRS), Université Paris-Sud, Université Paris-Saclay, UMR 9199, Neurodegenerative Diseases Laboratory, 18 Route du Panorama, F-92265 Fontenay-aux-Roses, France; b Commissariat à l’Énergie Atomique et aux Énergies Alternatives (CEA), Direction de la Recherche Fondamentale (DRF), Institut François Jacob, MIRCen, 18 Route du Panorama, F-92265 Fontenay-aux-Roses, France; c Inserm, Inserm UMR-S U1237, Normandie Univ, UNICAEN, GIP Cyceron, Caen, France; d Normandie University, UNICAEN, EPHE, INSERM, U1077, CHU de Caen, Neuropsychologie et Imagerie de la Mémoire Humaine, 14000 Caen, France; e Laboratoire de Psychopathologie et de Neuropsychologie, EA 2027, Université Paris 8, 2 Rue de la Liberté, 93000 St Denis, France

## Abstract

We present a dataset made of 3D digital brain templates and of an atlas of the gray mouse lemur (*Microcebus murinus*), a small prosimian primate of growing interest for studies of primate biology and evolution. A template image was constructed from *in vivo* magnetic resonance imaging (MRI) data of 34 animals. This template was then manually segmented into 40 cortical, 74 subcortical and 6 cerebro-spinal fluid (CSF) regions. Additionally, the dataset contains probability maps of gray matter, white matter and CSF. The template, manual segmentation and probability maps can be downloaded in NIfTI-1 format at https://www.nitrc.org/projects/mouselemuratlas. Further construction and validation details are given in “A 3D population-based brain atlas of the mouse lemur primate with examples of applications in aging studies and comparative anatomy” (Nadkarni et al., 2018) [1], which also presents applications of the atlas such as automatic assessment of regional age-associated cerebral atrophy and comparative neuroanatomy studies.

**Specifications table**

**Table:** 

Subject area	*Neuroscience*
More specific subject area	*Mouse lemur (Microcebus murinus) brain, MRI atlas*
Type of data	*Template, atlas and probabilistic maps for the mouse lemur brain*
	*Figure of the brain template and atlas.*
	*Figure of probabilistic maps for the mouse lemur brain*
	*Table of animals used for template creation*
	*Table with the list of segmented regions*
How data was acquired	in vivo *7T MRI (Agilent, Santa Clara, CA, USA)*
	*Template created with Sammba-MRI (*https://sammba-mri.github.io).
	Atlas created *using ITK-SNAP (*http://www.itksnap.org*)*
	*Probabilistic atlas created using SPM8 (*www.fil.ion.ucl.ac.uk/spm*) with the SPMMouse toolbox (*http://spmmouse.org*)*
Data format	*Analyzed (NIfTI-1 format)*
Experimental factors	*34 mouse lemurs (22 males and 12 females; age range 15–58 months)*
Experimental features	1. *A brain template was constructed from T2-weighted images of 34 mouse lemurs.* 2. *The template was segmented into 120 regions that covered the whole brain.* 3. *A probabilistic atlas was created from the initial brain template.*
Data source location	*Fontenay-aux-Roses, France*
Data accessibility	Data is with this article and available at NITRC:
	https://www.nitrc.org/projects/mouselemuratlas
Related research article	N.A. Nadkarni, S. Bougacha, C. Garin, M. Dhenain, J.L. Picq, A 3D population-based brain atlas of the mouse lemur primate with examples of applications in aging studies and comparative anatomy. NeuroImage, In press [1].

**Value of the data**

• This is the first publicly available whole brain template and atlas for the mouse lemur, an emergent model in neuroscience.
• The mouse lemur template and brain atlas can be used to study brain images of mouse lemurs recorded with various imaging modalities.
• A probabilistic atlas of the mouse lemur is also provided. It can be used as a prior for automatic segmentation studies.

## 1 Data

**Fig. 1:**
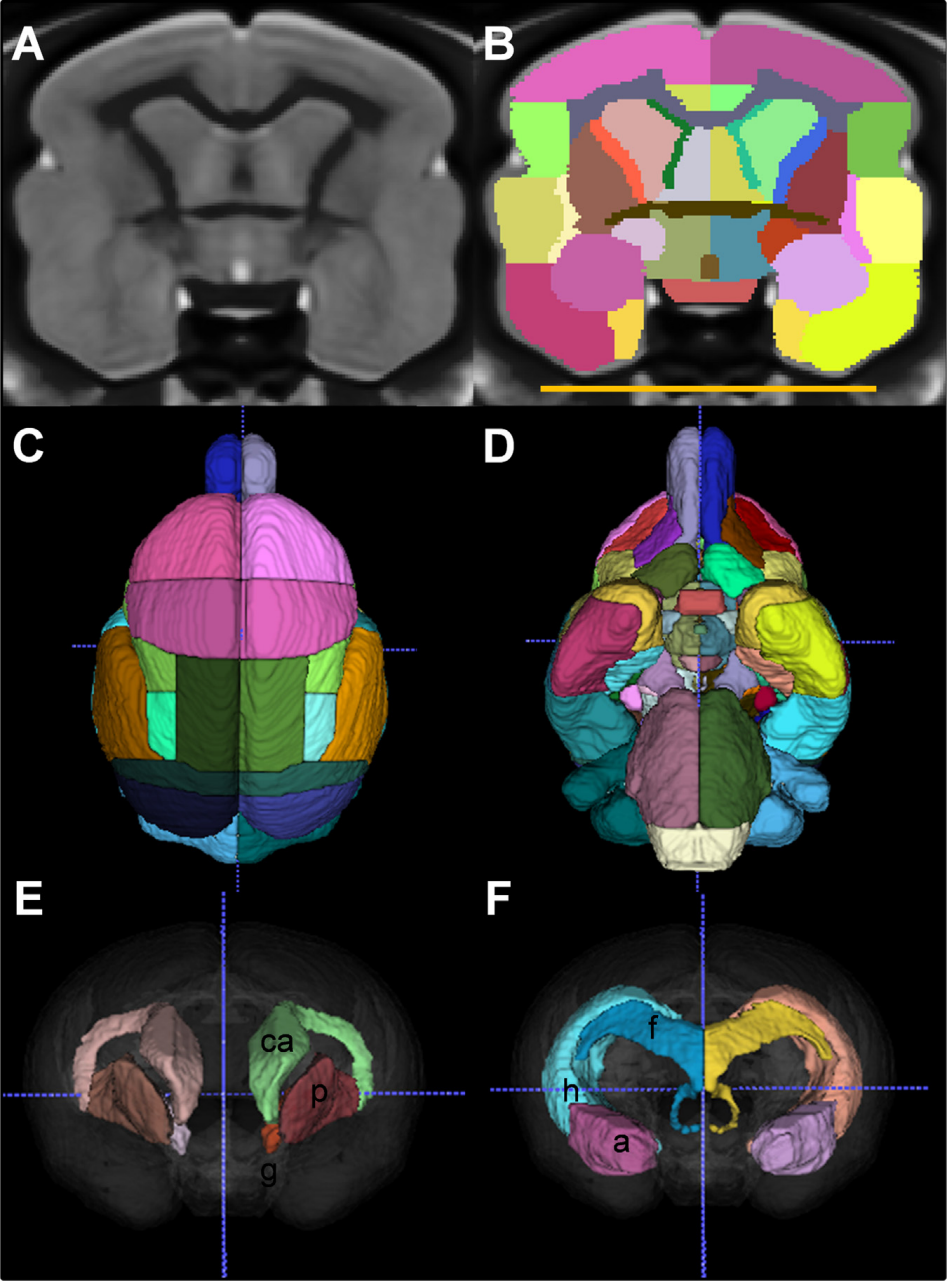
Labeling of the mouse lemur atlas. Brain structure delineations are shown in a coronal section (B) together with the corresponding template image (A). For clarity, the label marking surrounding CSF is not displayed. Superior (C) and inferior (D) views of the three-dimensional representation of the brain atlas. Anterior views of the basal ganglia (E) and limbic structures (F). Annotations: a = amygdala, ca = caudate nucleus, f = fornix, g = globus pallidus, h = hippocampus, p = putamen. Scale bar = 1 cm.

**Fig. 2:**
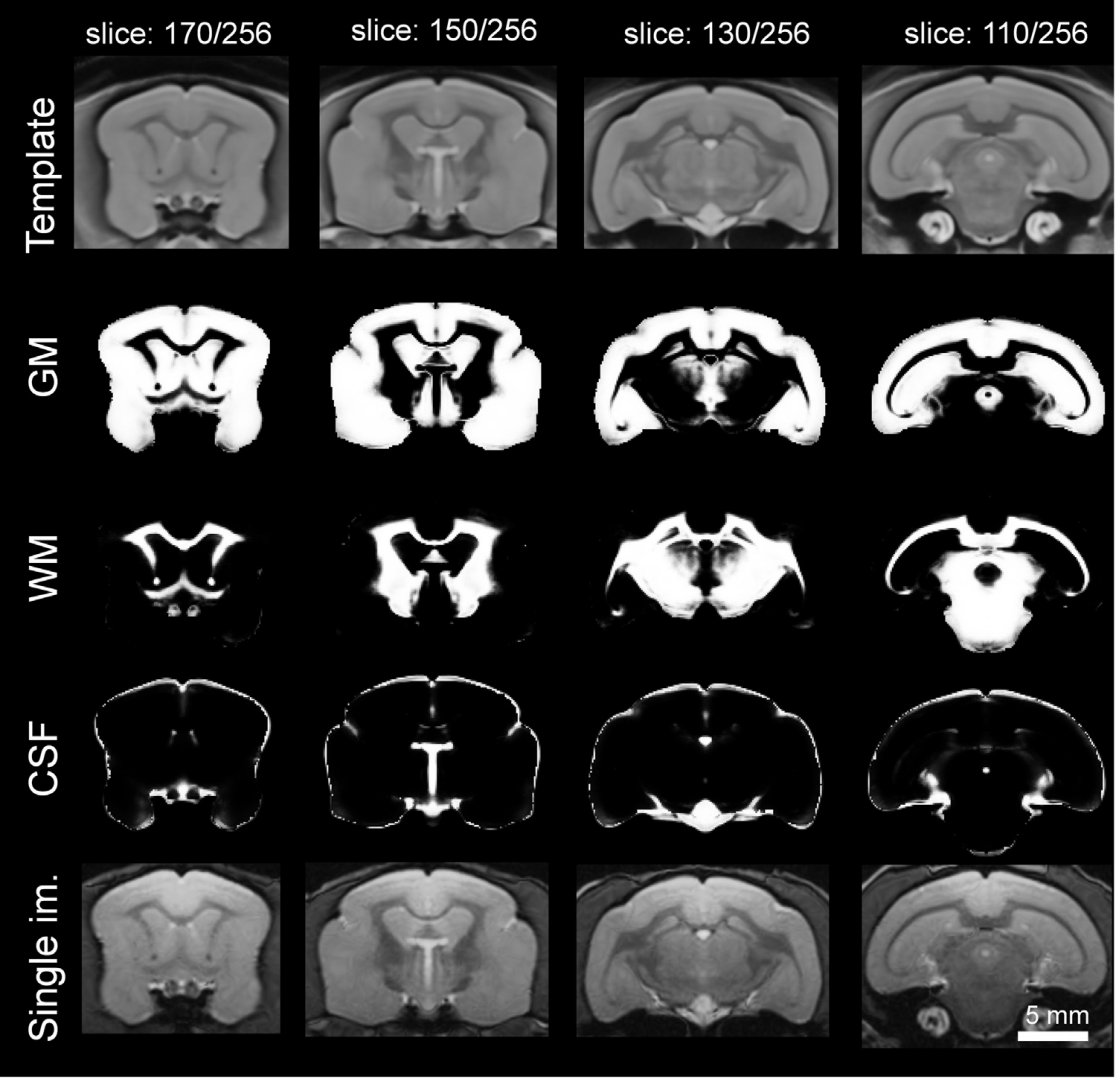
Template of the mouse lemur brain compared to probability maps and a representative image from a single animal. Scale bar: 5 mm.

MR images of the brain of 34 healthy young adult mouse lemurs (Table 1) were acquired in a 7 T scanner. 3D images of the whole brain were mutually registered to create a template (Fig. 1A). This template was used for manual segmentation (Fig. 1, Table 2) and to create probabilistic gray matter, white matter and CSF templates of the brain (Fig. 2). The templates and atlas are available as NIfTI volumes in an NITRC repository (https://www.nitrc.org/projects/mouselemuratlas). The dataset can be freely used for academic work upon citing this paper and [1].

**Table 1:** List of mouse lemurs used for atlas creation.

	Sex	Age (months)	Age (years)
147BCBB	M	28	2.3
190IAB	M	32	2.7
265B	M	32	2.7
190IC	M	32	2.7
967HACA	M	34	2.8
965MBGA	M	35	2.9
965MBFA	M	35	2.9
965MBFB	M	35	2.9
184CA	M	35	2.9
965MBIA	M	36	3.0
211DBA	M	39	3.2
169ABB	M	39	3.2
169ABC	M	39	3.2
259BB	M	40	3.3
199CBB	M	40	3.3
219G	M	40	3.3
189CBD	M	44	3.7
190IAA	M	46	3.8
260B	M	46	3.8
147BCBA	M	46	3.8
213ABA	M	47	3.9
153FBA	M	49	4.1
211EA	M	51	4.2
289BB	F	15	1.3
208CBF	F	18	1.5
288BC	F	18	1.5
310C	F	26	2.2
211AE	F	28	2.3
965MBFC	F	35	2.9
169BAB	F	35	2.9
184CB	F	36	3.0
967HACB	F	36	3.0
943GKBC	F	44	3.7
216B	F	58	4.8

**Table 2:** Labels of all brain structures used in the atlas. Note that label ID corresponds to voxel intensity in the atlas file that can be downloaded from https://www.nitrc.org/projects/mouselemuratlas.

**Label ID**	**Brain structure name**	**Label ID**	**Brain structure name**
1	hippocampal formation L	61	mammillary body L
2	hippocampal formation R	62	mammillary body R
3	amygdala L	63	hypophysis
4	amygdala R	64	pons L
5	caudate nucleus L	65	pons R
6	caudate nucleus R	66	nucleus accumbens L
7	stria terminalis L	67	nucleus accumbens R
8	stria terminalis R	68	basal forebrain nucleus L
9	CSF	69	basal forebrain nucleus R
10	anterior commissure	70	cerebellum L
11	arbor vitae of cerebellum L	71	cerebellum R
12	corpus callosum	72	arbor vitae of cerebellum R
13	fasciculus retroflexus L	73	cerebral aqueduct
14	fasciculus retroflexus R	74	posterior commissure
15	fornix L	75	cerebral cortex: area 6L
16	fornix R	76	cerebral cortex: area 4L
17	mamillo-thalamic tract L	77	cerebral cortex: area 8L
18	mamillo-thalamic tract R	78	cerebral cortex: area 1–3L
19	optic tract L	79	cerebral cortex: area 5L
20	optic tract R	80	cerebral cortex: area 7L
21	commissure of the inferior colliculus	81	cerebral cortex: area 13–16L
22	stria medullaris of the thalamus L	82	cerebral cortex: area 21L
23	stria medullaris of the thalamus R	83	cerebral cortex: area 22-(41–42) L
24	basal forebrain L	84	cerebral cortex: area 20L
25	basal forebrain R	85	cerebral cortex: area 18L
26	substantia nigra R	86	cerebral cortex: area 17L
27	substantia nigra L	87	cerebral cortex: area 28L
28	midbrain L	88	cerebral cortex: area 24L
29	midbrain R	89	cerebral cortex: area 23L
30	subthalamic nucleus L	90	cerebral cortex: area 30L
31	subthalamic nucleus R	91	cerebral cortex: area 26–29 (retrosplenial area) L
32	globus pallidus L	92	cerebral cortex: area 27L
33	globus pallidus R	93	cerebral cortex: prepyriform and periamygdalar areas L
34	putamen L	94	cerebral cortex: area 25L
35	putamen R	95	cerebral cortex: area 6R
36	habenula L	96	cerebral cortex: area 4R
37	habenula R	97	cerebral cortex: area 8R
38	septum L	98	cerebral cortex: area 1–3R
39	septum R	99	cerebral cortex: area 5R
40	claustrum L	100	cerebral cortex: area 7R
41	claustrum R	101	cerebral cortex: area 13–16R
42	hypothalamus L	102	cerebral cortex: area 21R
43	hypothalamus R	103	cerebral cortex: area 22-(41–42) R
44	thalamus L	104	cerebral cortex: area 20R
45	thalamus R	105	cerebral cortex: area 18R
46	central gray of the midbrain	106	cerebral cortex: area 17R
47	inferior colliculus L	107	cerebral cortex: area 28R
48	inferior colliculus R	108	cerebral cortex: area 24R
49	superior colliculus L	109	cerebral cortex: area 23R
50	superior colliculus R	110	cerebral cortex: area 30R
51	olfactory bulb L	111	cerebral cortex: area 26–29 (retrosplenial area) R
52	olfactory bulb R	112	cerebral cortex: area 27R
53	cerebral peduncle L	113	cerebral cortex: prepyriform and periamygdalar areas R
54	cerebral peduncle R	114	cerebral cortex: area 25R
55	internal capsule L	115	olfactory tubercle L
56	internal capsule R	116	olfactory tubercle R
57	lateral ventricle L	117	olfactory tract L
58	lateral ventricle R	118	olfactory tract R
59	third ventricle	119	optic chiasm
60	fourth ventricle	120	medulla

## 2 Experimental design, materials and methods

### 2.1 Animals

34 young to middle-aged adult mouse lemurs (22 males and 12 females) were used. Age range was 15–58 months, mean ± standard deviation 36.8 ± 9.2 months. Demographic information for these animals is provided in Table 1. The protocol was approved by the local ethics committee CEtEA-CEA DSV IdF (authorizations 201506051 736524 VI (APAFIS#778)) and followed the recommendations of the European Communities Council directive (2010/63/EU).

### 2.2 MR acquisition

One T2-weighted *in vivo* MRI scan was recorded for each animal. Animals were anesthetized by isoflurane (4% induction, 1–1.5% maintenance). Images were recorded using a 2D T2-weighted fast spin echo sequence (7 T Agilent system) using a four channel phased-array surface coil (Rapid Biomedical, Rimpar, Germany) actively decoupled from the transmitting birdcage probe (Rapid Biomedical, Rimpar, Germany), resolution 230 × 230 × 230 µm, TR/TE = 10,000/17.4 ms, RARE factor = 4, field of view (FOV) = 29.44 × 29.44 mm with a matrix (Mtx) = 128 × 128, 128 slices, number of averages (NA) = 6, acquisition duration 32 min.

### 2.3 Creation of the template

MR images from the 34 mouse lemurs were upsampled to 115 µm isotropic resolution. The template was generated using the function anats_to_common available within the sammba-mri python module (https://sammba-mri.github.io/generated/sammba.registration.anats_to_common.html#sammba.registration.anats_to_common). Most steps used tools from freely available AFNI software (https://afni.nimh.nih.gov/ [2], except for brain extraction which was done with RATS [3], [4]. First, head images were bias corrected. In a second step the brains were extracted and individual brain extracted image centers were shifted to the brain center of mass. Brains were then all rigid body aligned to a previous histological atlas of the mouse lemur brain [5] and the transform was then applied to the original heads. A first brain template (Template 1) was produced by averaging the aligned heads. A second template (Template 2) was created by using the previous rigid body registration step a second time to align the 34 centered brains to the first template. A third template (Template 3) was created by affine aligning the 34 centered brains to Template 2. A final template (Template 4) was created by executing four cycles of non-linear registration: the first one to affine Template 3, the other ones to templates of heads from the previous non-linear cycle, including initialization using the concatenated transforms of the previous cycles. Corrections for systematic biases in the non-linear transforms were applied after each cycle.

### 2.4 Segmentation of the MRI-based atlas

The template image was up-sampled to 91 µm isotropic resolution, then brain structures manually segmented in ITK-SNAP (http://www.itksnap.org [6];) according to published histological atlases [5], [7], [8]. Each structure was iteratively segmented slice by slice along the coronal, axial and sagittal orientations until the three-dimensional representation of the labelled structure was found to be smooth and non-jagged. Each structure was outlined bilaterally. In total, 120 regions including 40 cortical, 74 subcortical and 6 CSF regions were drawn (Fig. 1, labels of brain regions provided in Table 2). The names of the structures were based on the NeuroName ontology (http://www.braininfo.org [9]).

### 2.5 Tissue probability maps

Tissue probability maps that can be used for brain morphometry analyses were created using SPM8 (www.fil.ion.ucl.ac.uk/spm) with the SPMMouse toolbox (http://spmmouse.org) [10], [11]. MR images from the 34 animals of the study were registered to an SPM template of the mouse lemur brain [11]. Affine registration registered the images to control for different head positions, scanner geometry and overall brain size. Then unified segmentation iteratively warped the data whilst correcting for signal inhomogeneity. The images of the rigidly-aligned brains of each animal were then segmented using a k-means algorithm [12] with 4 segments: background, GM, WM, and CSF. These maps were then averaged across individuals separately for each tissue type to produce mean GM, WM and CSF tissue probability maps. These probabilistic maps were manually edited to correct for mislabeling of CSF as GM or WM voxels due to partial volume effects, in particular around edges of the brain. They were also masked using masks derived from the segmented atlas, to conserve only brain and CSF structures (Fig. 2).
