# Supplementary material for: Digital templates and brain atlas dataset for the mouse lemur primate
Source: Data Brief. 2018 Oct 25;21:1178–85. doi: 10.1016/j.dib.2018.10.067 (PMC6230976; doi:10.1016/j.dib.2018.10.067)
Supplement: Supplementary file 1 — Supplementary material [file mmc1.docx]

# Digital templates and brain atlas of the mouse lemur primate

# Disclosure Statement

**1a. Actual or potential conflicts of interest**

Nachiket A. Nadkarni: No actual or potential conflicts of interest

Salma Bougacha: No actual or potential conflicts of interest

Clément Garin: No actual or potential conflicts of interest

Marc Dhenain: No actual or potential conflicts of interest

Jean-Luc Picq: No actual or potential conflicts of interest

**1b. Contracts from author's institution in relation with this research through which author's institution or any other organization may stand to gain financially now or in the future.**

Nachiket A. Nadkarni: None

Salma Bougacha: None

Clément Garin: None

Marc Dhenain: None

Jean-Luc Picq: None

**1c. Any other agreements of authors or their institutions that could be seen as involving a financial interest in this work.**

Nachiket A. Nadkarni: None

Salma Bougacha: None

Clément Garin: None

Marc Dhenain: None

Jean-Luc Picq: None
